# *S*-Benzyl­thio­uronium 3-nitro­benzene­sulfonate

**DOI:** 10.1107/S1600536808016115

**Published:** 2008-06-07

**Authors:** Hoong-Kun Fun, Samuel Robinson Jebas, Ibrahim Abdul Razak, E. Deepak D’Silva, P. S. Patil, S. M. Dharmaprakash

**Affiliations:** a X-ray Crystallography Unit, School of Physics, Universiti Sains Malaysia, 11800 USM, Penang, Malaysia; b Department of Studies in Physics, Mangalore University, Mangalagangotri, Mangalore 574 199, India

## Abstract

In the title compound, C_8_H_11_N_2_S^+^·C_6_H_4_NO_5_S^−^, the asymmetric unit is composed of two crystallographically independent *S*-benzyl­thio­uronium cations and two independent nitro­benzene­sulfonate anions. An intra­molecular hydrogen bond generates an *S*(5)*S*(5) ring motif. The crystal packing is stabilized by intra­molecular C—H⋯O and inter­molecular C—H⋯O, N—H⋯O and N—H⋯S hydrogen bonds which, along with short S⋯O [3.034 (2) Å] and N⋯O [2.796 (3) Å] contacts, form a two-dimensional network parallel to the *ab* plane.

## Related literature

For related literature on nonlinear optical materials, see: Chantrapromma *et al.* (2005 ▶, 2006 ▶); Fun *et al.* (2006 ▶); Patil, Dharmaprakash *et al.* (2007 ▶); Patil, Fun *et al.* (2007 ▶). For bond-length data, see: Allen *et al.* (1987 ▶). For graph-set analysis of hydrogen bonding, see: Bernstein *et al.* (1995 ▶).
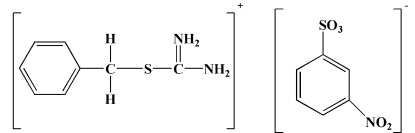


## Experimental

### 

#### Crystal data

C_8_H_11_N_2_S^+^·C_6_H_4_NO_5_S^−^
*M* *_r_* = 369.41
Triclinic, 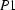

*a* = 6.0397 (1) Å
*b* = 7.7856 (1) Å
*c* = 17.4680 (2) Å
α = 81.366 (1)°
β = 89.322 (1)°
γ = 87.057 (1)°
*V* = 811.01 (2) Å^3^
*Z* = 2
Mo *K*α radiation
μ = 0.36 mm^−1^
*T* = 100.0 (1) K
0.32 × 0.19 × 0.04 mm

#### Data collection

Bruker SMART APEXII CCD area-detector diffractometer
Absorption correction: multi-scan (*SADABS*; Bruker, 2005 ▶) *T*_min_ = 0.894, *T*_max_ = 0.986
19026 measured reflections
8874 independent reflections
7378 reflections with *I* > 2σ(*I*)
*R*_int_ = 0.043

#### Refinement

*R*[*F* ^2^ > 2σ(*F* ^2^)] = 0.044
*wR*(*F* ^2^) = 0.112
*S* = 1.03
8874 reflections
433 parameters
3 restraints
H-atom parameters constrained
Δρ_max_ = 0.38 e Å^−3^
Δρ_min_ = −0.32 e Å^−3^
Absolute structure: Flack (1983 ▶), 4108 Friedel pairs
Flack parameter: −0.03 (5)

### 

Data collection: *APEX2* (Bruker, 2005 ▶); cell refinement: *APEX2*; data reduction: *SAINT* (Bruker, 2005 ▶); program(s) used to solve structure: *SHELXTL* (Sheldrick, 2008 ▶); program(s) used to refine structure: *SHELXTL*; molecular graphics: *SHELXTL*; software used to prepare material for publication: *SHELXTL* and *PLATON* (Spek, 2003 ▶).

## Supplementary Material

Crystal structure: contains datablocks global, I. DOI: 10.1107/S1600536808016115/su2055sup1.cif

Structure factors: contains datablocks I. DOI: 10.1107/S1600536808016115/su2055Isup2.hkl

Additional supplementary materials:  crystallographic information; 3D view; checkCIF report

## Figures and Tables

**Table 1:** Hydrogen-bond geometry (Å, °)

*D*—H⋯*A*	*D*—H	H⋯*A*	*D*⋯*A*	*D*—H⋯*A*
N3—H1N3⋯O4^i^	0.86	1.97	2.796 (3)	162
N3—H1N3⋯S1^i^	0.86	2.84	3.694 (3)	170
N3—H2N3⋯O3^ii^	0.86	2.05	2.898 (3)	171
N4—H1N4⋯O3^i^	0.86	2.26	3.080 (3)	160
N4—H2N4⋯O5^iii^	0.86	2.37	3.126 (3)	146
N5—H1N5⋯O10^i^	0.86	1.91	2.764 (3)	176
N5—H1N5⋯S2^i^	0.86	2.85	3.642 (3)	154
N5—H2N5⋯O9^ii^	0.86	1.94	2.783 (3)	168
N6—H1N6⋯O1^iii^	0.86	2.27	3.072 (3)	156
N6—H2N6⋯O8^iii^	0.86	2.07	2.787 (3)	141
C6—H6*A*⋯O4	0.93	2.56	2.900 (3)	102
C8—H8*A*⋯O10	0.93	2.57	2.896 (4)	101
C19—H19*B*⋯O4^iii^	0.97	2.51	3.331 (4)	142
C27—H27*B*⋯O8^iii^	0.97	2.53	3.259 (4)	132

Symmetry codes: (i) 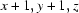; (ii) 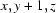; (iii) 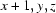.

**Fig. 1.**
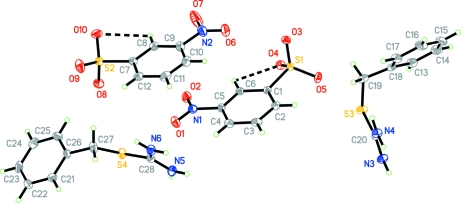
The molecular structure of compound (I) showing 50% probability displacement ellipsoids and the atom numbering scheme.

**Fig. 2.**
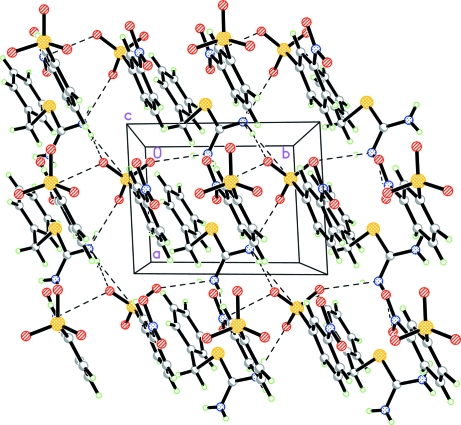
The crystal packing of compound (I) viewed down the c axis, showing the two-dimensional network. Short intra and intermolecular contacts and hydrogen bonds are shown as dashed lines.

## supplementary crystallographic information

### Comment

In continuation of our research on nonlinear optical (NLO) materials (Chantrapromma *et al.*, 2005, 2006; Fun *et al.*, 2006; Patil, Dharmaprakash *et al.* (2007); Patil, Fun *et al.* (2007), the crystal structure determination of the title compound, (I), was undertaken in order to obtain detailed information on its three-dimensional structure and crystal packing. Since the title compound crystallizes in a non-centrosymmetric space group, it should exhibit second-order nonlinear optical properties.

In the asymmetric unit of (I) there are two crystallographically independent s-benzylthiuronium cations and two independent m-nitrobenzene sulfonate anions (Fig. 1). Bond lengths and angles are found to have normal values (Allen *et al.*, 1987). The dihedral angle formed by the mean plane of ring (C13–C18) with the mean planes through rings (C21–C26), (C1–C6) and (C7–C12) are 57.6 (2)°, 49.9 (2)° and 46.98 (1)°, respectively. The dihedral angle formed by the mean planes of rings (C1–C6) and (C7–C12) are 3.07 (1)°, indicating that they are almost coplanar.

An intramolecular hydrogen bond generates a S(5)S(5) ring motif (Bernstein *et al.*, 1995). The crystal packing (Fig. 2) is stabilized by intramolecular C—H···O, intermolecular C—H···O, N—H···O and N—H···S hydrogen bonds which together with short S···O and N···O contacts, in the range 2.796 (3)–3.282 (3) Å [symmetry code: x, 1+y, z; 1+x, 1+y, z], form a two-dimensional network parrallel to the ab plane.

### Experimental

Compound (I) was synthesized by mixing solutions of the sodium salt of *m*-nitrobenzene sulfonic acid (0.5 g) in 5 ml of distilled water with 5 drops of 0.1 N HCl and *S*-benzylthiuronium chloride (1 g) in 5 ml of distilled water. The mixing immediately yielded a precipitate when the reaction container was placed in ice cold water. The resulting precipitate was filtered and dried. Crystals suitable for single-crystal X-ray diffraction were grown by slow evaporation of an ethanol solution at room temperature.

### Refinement

H atoms were positioned geometrically [C—H = 0.93 Å; N—H = 0.86 Å and CH_2_ = 0.97 Å] and refined using a riding model with *U*_iso_(H) = 1.2*U*_eq_(C, N).

### Crystal data

**Table:**

C_8_H_11_N_2_S^+^·C_6_H_4_NO_5_S^–^	*Z* = 2
*M**_r_* = 369.41	*F*_000_ = 384
Triclinic, *P*1	*D*_x_ = 1.513 Mg m^−^^3^
Hall symbol: P 1	Mo *K*α radiation λ = 0.71073 Å
*a* = 6.0397 (1) Å	Cell parameters from 4354 reflections
*b* = 7.7856 (1) Å	θ = 2.8–34.6º
*c* = 17.4680 (2) Å	µ = 0.36 mm^−^^1^
α = 81.366 (1)º	*T* = 100.0 (1) K
β = 89.322 (1)º	Plate, colourless
γ = 87.057 (1)º	0.32 × 0.19 × 0.04 mm
*V* = 811.006 (19) Å^3^	

### Data collection

**Table:**

Bruker SMART APEXII CCD area-detector diffractometer	8874 independent reflections
Radiation source: fine-focus sealed tube	7378 reflections with *I* > 2σ(*I*)
Monochromator: graphite	*R*_int_ = 0.043
*T* = 100.0(1) K	θ_max_ = 30.1º
φ and ω scans	θ_min_ = 1.2º
Absorption correction: multi-scan(SADABS; Bruker, 2005)	*h* = −7→8
*T*_min_ = 0.894, *T*_max_ = 0.986	*k* = −10→10
19026 measured reflections	*l* = −24→24

### Refinement

**Table:**

Refinement on *F*^2^	Hydrogen site location: inferred from neighbouring sites
Least-squares matrix: full	H-atom parameters constrained
*R*[*F*^2^ > 2σ(*F*^2^)] = 0.044	*w* = 1/[σ^2^(*F*_o_^2^) + (0.0534*P*)^2^] where *P* = (*F*_o_^2^ + 2*F*_c_^2^)/3
*wR*(*F*^2^) = 0.112	(Δ/σ)_max_ < 0.001
*S* = 1.03	Δρ_max_ = 0.38 e Å^−^^3^
8874 reflections	Δρ_min_ = −0.32 e Å^−^^3^
433 parameters	Extinction correction: none
3 restraints	Absolute structure: Flack (1983), with how many Friedel pairs?
Primary atom site location: structure-invariant direct methods	Flack parameter: −0.03 (5)
Secondary atom site location: difference Fourier map	

### Special details

**Table:**

Experimental. The data was collected with the Oxford Cyrosystem Cobra low-temperature attachment.
Geometry. All e.s.d.'s (except the e.s.d. in the dihedral angle between two l.s. planes) are estimated using the full covariance matrix. The cell e.s.d.'s are taken into account individually in the estimation of e.s.d.'s in distances, angles and torsion angles; correlations between e.s.d.'s in cell parameters are only used when they are defined by crystal symmetry. An approximate (isotropic) treatment of cell e.s.d.'s is used for estimating e.s.d.'s involving l.s. planes.
Refinement. Refinement of *F*^2^ against ALL reflections. The weighted *R*-factor *wR* and goodness of fit *S* are based on *F*^2^, conventional *R*-factors *R* are based on *F*, with *F* set to zero for negative *F*^2^. The threshold expression of *F*^2^ > σ(*F*^2^) is used only for calculating *R*-factors(gt) *etc*. and is not relevant to the choice of reflections for refinement. *R*-factors based on *F*^2^ are statistically about twice as large as those based on *F*, and *R*- factors based on ALL data will be even larger.

### Fractional atomic coordinates and isotropic or equivalent isotropic displacement parameters (Å^2^)

**Table:**

	*x*	*y*	*z*	*U*_iso_*/*U*_eq_	
S1	0.37546 (10)	0.53723 (8)	0.82205 (4)	0.01444 (14)	
O1	0.4811 (4)	0.4607 (3)	0.47221 (12)	0.0252 (5)	
O2	0.1931 (4)	0.4114 (3)	0.54531 (12)	0.0241 (5)	
O3	0.4577 (3)	0.3635 (3)	0.85663 (11)	0.0180 (4)	
O4	0.1377 (3)	0.5430 (3)	0.80849 (11)	0.0184 (4)	
O5	0.4448 (3)	0.6737 (3)	0.86311 (12)	0.0206 (4)	
N1	0.3820 (4)	0.4617 (3)	0.53419 (14)	0.0193 (5)	
C1	0.4988 (4)	0.5723 (3)	0.72854 (15)	0.0147 (5)	
C2	0.7020 (4)	0.6478 (4)	0.71677 (16)	0.0167 (5)	
H2A	0.7710	0.6903	0.7569	0.020*	
C3	0.8017 (5)	0.6590 (4)	0.64337 (18)	0.0207 (6)	
H3A	0.9384	0.7085	0.6351	0.025*	
C4	0.7004 (5)	0.5977 (4)	0.58323 (17)	0.0184 (6)	
H4A	0.7673	0.6044	0.5347	0.022*	
C5	0.4953 (5)	0.5255 (4)	0.59719 (16)	0.0163 (5)	
C6	0.3912 (5)	0.5112 (3)	0.66886 (15)	0.0154 (5)	
H6A	0.2539	0.4623	0.6767	0.018*	
S2	0.31950 (11)	−0.08507 (8)	0.41082 (4)	0.01834 (15)	
O6	0.5275 (5)	0.1046 (3)	0.73759 (13)	0.0379 (6)	
O7	0.2256 (5)	0.0097 (4)	0.69863 (14)	0.0493 (8)	
O8	0.1789 (4)	0.0611 (3)	0.37490 (12)	0.0231 (5)	
O9	0.4738 (4)	−0.1484 (3)	0.35681 (13)	0.0301 (5)	
O10	0.1907 (4)	−0.2203 (3)	0.45482 (13)	0.0282 (5)	
N2	0.4156 (5)	0.0558 (4)	0.68796 (15)	0.0285 (6)	
C7	0.4798 (5)	−0.0078 (4)	0.48202 (16)	0.0168 (5)	
C8	0.3892 (5)	−0.0087 (4)	0.55576 (17)	0.0185 (6)	
H8A	0.2493	−0.0502	0.5677	0.022*	
C9	0.5124 (5)	0.0534 (4)	0.61044 (17)	0.0215 (6)	
C10	0.7218 (5)	0.1167 (4)	0.59464 (18)	0.0227 (6)	
H10A	0.8017	0.1578	0.6327	0.027*	
C11	0.8082 (5)	0.1169 (4)	0.52112 (19)	0.0239 (6)	
H11A	0.9479	0.1590	0.5094	0.029*	
C12	0.6880 (5)	0.0546 (4)	0.46421 (17)	0.0195 (6)	
H12A	0.7471	0.0549	0.4148	0.023*	
S3	0.77990 (11)	0.92863 (9)	0.89784 (5)	0.02185 (17)	
N3	0.9177 (4)	1.2377 (3)	0.85658 (14)	0.0211 (5)	
H1N3	1.0112	1.3173	0.8448	0.025*	
H2N3	0.7778	1.2643	0.8544	0.025*	
N4	1.1997 (4)	1.0283 (3)	0.88215 (14)	0.0199 (5)	
H1N4	1.2973	1.1048	0.8708	0.024*	
H2N4	1.2408	0.9209	0.8963	0.024*	
C13	0.8638 (5)	0.8126 (4)	1.08658 (18)	0.0229 (6)	
H13A	0.7231	0.8611	1.0730	0.027*	
C14	0.9388 (6)	0.8053 (4)	1.16156 (19)	0.0292 (7)	
H14A	0.8479	0.8490	1.1982	0.035*	
C15	1.1470 (6)	0.7337 (4)	1.18259 (18)	0.0294 (7)	
H15A	1.1961	0.7290	1.2332	0.035*	
C16	1.2827 (6)	0.6688 (4)	1.12789 (19)	0.0274 (7)	
H16A	1.4234	0.6208	1.1417	0.033*	
C17	1.2086 (5)	0.6754 (4)	1.05282 (17)	0.0217 (6)	
H17A	1.3000	0.6312	1.0164	0.026*	
C18	0.9993 (5)	0.7473 (4)	1.03121 (17)	0.0178 (6)	
C19	0.9156 (5)	0.7358 (4)	0.95108 (18)	0.0216 (6)	
H19A	0.8127	0.6432	0.9556	0.026*	
H19B	1.0406	0.7015	0.9204	0.026*	
C20	0.9890 (5)	1.0752 (4)	0.87791 (15)	0.0165 (5)	
S4	0.70823 (11)	0.42238 (9)	0.29521 (4)	0.02029 (15)	
N5	0.8675 (4)	0.6477 (3)	0.37270 (14)	0.0205 (5)	
H1N5	0.9647	0.6932	0.3980	0.025*	
H2N5	0.7379	0.6980	0.3650	0.025*	
N6	1.1136 (4)	0.4218 (3)	0.35682 (14)	0.0209 (5)	
H1N6	1.2132	0.4650	0.3819	0.025*	
H2N6	1.1425	0.3262	0.3388	0.025*	
C21	0.7614 (5)	0.3251 (4)	0.09917 (17)	0.0211 (6)	
H21A	0.8905	0.3852	0.0900	0.025*	
C22	0.6228 (5)	0.3107 (4)	0.03818 (17)	0.0241 (6)	
H22A	0.6595	0.3609	−0.0118	0.029*	
C23	0.4305 (5)	0.2224 (4)	0.05098 (18)	0.0258 (7)	
H23A	0.3378	0.2128	0.0099	0.031*	
C24	0.3766 (5)	0.1484 (4)	0.12517 (19)	0.0266 (7)	
H24A	0.2463	0.0897	0.1341	0.032*	
C25	0.5157 (5)	0.1608 (4)	0.18650 (17)	0.0228 (6)	
H25A	0.4794	0.1091	0.2362	0.027*	
C26	0.7088 (5)	0.2503 (4)	0.17390 (16)	0.0180 (6)	
C27	0.8588 (5)	0.2713 (4)	0.23962 (17)	0.0242 (6)	
H27A	0.9976	0.3182	0.2200	0.029*	
H27B	0.8905	0.1601	0.2718	0.029*	
C28	0.9169 (5)	0.5019 (4)	0.34598 (16)	0.0175 (6)	

### Atomic displacement parameters (Å^2^)

**Table:**

	*U*^11^	*U*^22^	*U*^33^	*U*^12^	*U*^13^	*U*^23^
S1	0.0132 (3)	0.0156 (3)	0.0147 (3)	−0.0010 (2)	−0.0006 (2)	−0.0028 (2)
O1	0.0274 (12)	0.0322 (12)	0.0163 (10)	0.0034 (9)	0.0009 (9)	−0.0061 (9)
O2	0.0218 (11)	0.0285 (12)	0.0236 (11)	−0.0070 (9)	−0.0016 (9)	−0.0066 (9)
O3	0.0197 (10)	0.0164 (10)	0.0169 (9)	0.0018 (8)	−0.0002 (8)	0.0000 (8)
O4	0.0138 (10)	0.0201 (10)	0.0209 (10)	−0.0010 (8)	0.0001 (8)	−0.0019 (8)
O5	0.0202 (10)	0.0223 (10)	0.0222 (10)	−0.0061 (8)	0.0008 (8)	−0.0111 (8)
N1	0.0229 (14)	0.0157 (12)	0.0188 (12)	0.0024 (10)	−0.0025 (10)	−0.0019 (9)
C1	0.0144 (13)	0.0126 (13)	0.0167 (13)	0.0009 (10)	−0.0022 (10)	−0.0018 (10)
C2	0.0130 (13)	0.0167 (13)	0.0199 (13)	−0.0007 (10)	−0.0032 (10)	−0.0012 (11)
C3	0.0128 (13)	0.0199 (14)	0.0273 (15)	−0.0018 (10)	0.0006 (11)	0.0034 (12)
C4	0.0167 (14)	0.0195 (14)	0.0182 (13)	0.0018 (11)	0.0020 (11)	−0.0008 (11)
C5	0.0176 (13)	0.0149 (13)	0.0166 (12)	0.0015 (10)	−0.0048 (10)	−0.0031 (10)
C6	0.0141 (13)	0.0144 (13)	0.0170 (13)	−0.0010 (10)	−0.0017 (10)	−0.0002 (10)
S2	0.0198 (4)	0.0174 (3)	0.0176 (3)	0.0013 (3)	−0.0044 (3)	−0.0023 (3)
O6	0.0591 (18)	0.0348 (14)	0.0216 (12)	−0.0018 (12)	−0.0106 (11)	−0.0094 (10)
O7	0.0337 (15)	0.092 (2)	0.0243 (13)	−0.0050 (15)	0.0061 (11)	−0.0138 (14)
O8	0.0235 (11)	0.0209 (11)	0.0232 (10)	0.0045 (8)	−0.0055 (9)	0.0004 (9)
O9	0.0310 (13)	0.0378 (13)	0.0224 (11)	0.0120 (10)	−0.0038 (9)	−0.0118 (10)
O10	0.0330 (13)	0.0221 (11)	0.0284 (11)	−0.0104 (9)	−0.0110 (10)	0.0035 (9)
N2	0.0390 (18)	0.0263 (14)	0.0199 (13)	0.0067 (12)	−0.0021 (12)	−0.0052 (11)
C7	0.0169 (14)	0.0150 (13)	0.0175 (13)	0.0034 (10)	−0.0031 (10)	−0.0003 (10)
C8	0.0172 (14)	0.0171 (13)	0.0205 (13)	0.0024 (11)	−0.0019 (11)	−0.0019 (11)
C9	0.0232 (16)	0.0201 (15)	0.0206 (14)	0.0059 (11)	−0.0056 (11)	−0.0036 (11)
C10	0.0206 (15)	0.0202 (15)	0.0276 (15)	0.0037 (11)	−0.0091 (12)	−0.0055 (12)
C11	0.0149 (14)	0.0199 (15)	0.0363 (17)	−0.0018 (11)	−0.0032 (12)	−0.0021 (13)
C12	0.0178 (14)	0.0164 (14)	0.0235 (14)	0.0011 (11)	−0.0009 (11)	−0.0013 (11)
S3	0.0150 (4)	0.0199 (4)	0.0292 (4)	−0.0045 (3)	−0.0034 (3)	0.0025 (3)
N3	0.0147 (12)	0.0189 (12)	0.0294 (13)	−0.0045 (9)	−0.0001 (10)	−0.0012 (10)
N4	0.0142 (12)	0.0211 (13)	0.0241 (12)	−0.0025 (9)	0.0002 (10)	−0.0016 (10)
C13	0.0206 (15)	0.0208 (15)	0.0268 (15)	−0.0007 (11)	0.0053 (12)	−0.0024 (12)
C14	0.0363 (19)	0.0219 (16)	0.0287 (16)	−0.0027 (13)	0.0097 (14)	−0.0010 (13)
C15	0.038 (2)	0.0278 (18)	0.0200 (15)	−0.0090 (14)	−0.0013 (13)	0.0053 (13)
C16	0.0228 (16)	0.0265 (17)	0.0298 (17)	−0.0019 (12)	−0.0054 (13)	0.0067 (13)
C17	0.0192 (15)	0.0220 (15)	0.0224 (14)	0.0022 (11)	0.0039 (12)	−0.0002 (12)
C18	0.0176 (14)	0.0130 (13)	0.0224 (14)	−0.0041 (10)	0.0024 (11)	−0.0004 (11)
C19	0.0195 (15)	0.0150 (14)	0.0298 (16)	−0.0016 (11)	0.0014 (12)	−0.0017 (12)
C20	0.0156 (13)	0.0207 (14)	0.0136 (12)	−0.0031 (10)	0.0007 (10)	−0.0027 (10)
S4	0.0170 (4)	0.0252 (4)	0.0202 (3)	−0.0003 (3)	−0.0022 (3)	−0.0082 (3)
N5	0.0177 (12)	0.0222 (13)	0.0228 (12)	−0.0013 (9)	−0.0009 (10)	−0.0067 (10)
N6	0.0187 (13)	0.0233 (13)	0.0212 (12)	0.0006 (10)	−0.0056 (10)	−0.0050 (10)
C21	0.0222 (15)	0.0188 (14)	0.0224 (14)	−0.0025 (11)	0.0019 (12)	−0.0027 (12)
C22	0.0329 (18)	0.0236 (16)	0.0157 (13)	−0.0003 (13)	−0.0014 (12)	−0.0027 (12)
C23	0.0283 (17)	0.0249 (16)	0.0265 (16)	0.0023 (13)	−0.0125 (13)	−0.0116 (13)
C24	0.0242 (16)	0.0241 (16)	0.0335 (17)	−0.0064 (12)	−0.0009 (13)	−0.0087 (13)
C25	0.0269 (16)	0.0221 (15)	0.0185 (14)	−0.0005 (12)	0.0036 (12)	−0.0001 (12)
C26	0.0215 (15)	0.0182 (14)	0.0149 (12)	0.0013 (11)	−0.0023 (11)	−0.0047 (11)
C27	0.0256 (16)	0.0286 (16)	0.0197 (14)	0.0069 (12)	−0.0035 (12)	−0.0103 (12)
C28	0.0163 (14)	0.0215 (15)	0.0144 (12)	−0.0026 (11)	0.0008 (11)	−0.0011 (11)

### Geometric parameters (Å, °)

**Table:**

S1—O5	1.449 (2)	N4—C20	1.306 (4)
S1—O4	1.456 (2)	N4—H1N4	0.8600
S1—O3	1.461 (2)	N4—H2N4	0.8600
S1—C1	1.776 (3)	C13—C14	1.383 (5)
O1—N1	1.232 (3)	C13—C18	1.396 (4)
O2—N1	1.229 (3)	C13—H13A	0.9300
N1—C5	1.464 (4)	C14—C15	1.380 (5)
C1—C2	1.388 (4)	C14—H14A	0.9300
C1—C6	1.390 (4)	C15—C16	1.387 (5)
C2—C3	1.403 (4)	C15—H15A	0.9300
C2—H2A	0.9300	C16—C17	1.383 (4)
C3—C4	1.379 (4)	C16—H16A	0.9300
C3—H3A	0.9300	C17—C18	1.389 (4)
C4—C5	1.390 (4)	C17—H17A	0.9300
C4—H4A	0.9300	C18—C19	1.510 (4)
C5—C6	1.386 (4)	C19—H19A	0.9700
C6—H6A	0.9300	C19—H19B	0.9700
S2—O9	1.440 (2)	S4—C28	1.740 (3)
S2—O8	1.452 (2)	S4—C27	1.835 (3)
S2—O10	1.460 (2)	N5—C28	1.310 (4)
S2—C7	1.778 (3)	N5—H1N5	0.8600
O6—N2	1.222 (4)	N5—H2N5	0.8600
O7—N2	1.224 (4)	N6—C28	1.315 (4)
N2—C9	1.471 (4)	N6—H1N6	0.8600
C7—C12	1.386 (4)	N6—H2N6	0.8600
C7—C8	1.393 (4)	C21—C22	1.384 (4)
C8—C9	1.376 (4)	C21—C26	1.387 (4)
C8—H8A	0.9300	C21—H21A	0.9300
C9—C10	1.390 (4)	C22—C23	1.379 (5)
C10—C11	1.380 (5)	C22—H22A	0.9300
C10—H10A	0.9300	C23—C24	1.379 (4)
C11—C12	1.396 (4)	C23—H23A	0.9300
C11—H11A	0.9300	C24—C25	1.387 (4)
C12—H12A	0.9300	C24—H24A	0.9300
S3—C20	1.742 (3)	C25—C26	1.388 (4)
S3—C19	1.806 (3)	C25—H25A	0.9300
N3—C20	1.316 (4)	C26—C27	1.504 (4)
N3—H1N3	0.8600	C27—H27A	0.9700
N3—H2N3	0.8600	C27—H27B	0.9700
			
O5—S1—O4	113.68 (12)	C14—C13—C18	120.0 (3)
O5—S1—O3	113.38 (12)	C14—C13—H13A	120.0
O4—S1—O3	111.39 (12)	C18—C13—H13A	120.0
O5—S1—C1	107.07 (12)	C15—C14—C13	120.7 (3)
O4—S1—C1	105.35 (12)	C15—C14—H14A	119.6
O3—S1—C1	105.17 (12)	C13—C14—H14A	119.6
O2—N1—O1	122.9 (2)	C14—C15—C16	119.6 (3)
O2—N1—C5	118.8 (2)	C14—C15—H15A	120.2
O1—N1—C5	118.3 (2)	C16—C15—H15A	120.2
C2—C1—C6	121.3 (3)	C17—C16—C15	120.0 (3)
C2—C1—S1	121.1 (2)	C17—C16—H16A	120.0
C6—C1—S1	117.5 (2)	C15—C16—H16A	120.0
C1—C2—C3	118.9 (3)	C16—C17—C18	120.7 (3)
C1—C2—H2A	120.5	C16—C17—H17A	119.6
C3—C2—H2A	120.5	C18—C17—H17A	119.6
C4—C3—C2	121.1 (3)	C17—C18—C13	119.0 (3)
C4—C3—H3A	119.4	C17—C18—C19	119.0 (3)
C2—C3—H3A	119.4	C13—C18—C19	121.7 (3)
C3—C4—C5	118.1 (3)	C18—C19—S3	117.6 (2)
C3—C4—H4A	121.0	C18—C19—H19A	107.9
C5—C4—H4A	121.0	S3—C19—H19A	107.9
C6—C5—C4	122.7 (3)	C18—C19—H19B	107.9
C6—C5—N1	117.9 (2)	S3—C19—H19B	107.9
C4—C5—N1	119.4 (2)	H19A—C19—H19B	107.2
C5—C6—C1	117.8 (3)	N4—C20—N3	122.3 (3)
C5—C6—H6A	121.1	N4—C20—S3	123.1 (2)
C1—C6—H6A	121.1	N3—C20—S3	114.6 (2)
O9—S2—O8	112.40 (13)	C28—S4—C27	103.46 (14)
O9—S2—O10	113.93 (15)	C28—N5—H1N5	120.0
O8—S2—O10	111.89 (14)	C28—N5—H2N5	120.0
O9—S2—C7	106.75 (14)	H1N5—N5—H2N5	120.0
O8—S2—C7	106.93 (13)	C28—N6—H1N6	120.0
O10—S2—C7	104.21 (13)	C28—N6—H2N6	120.0
O6—N2—O7	123.8 (3)	H1N6—N6—H2N6	120.0
O6—N2—C9	118.6 (3)	C22—C21—C26	120.4 (3)
O7—N2—C9	117.6 (3)	C22—C21—H21A	119.8
C12—C7—C8	120.9 (3)	C26—C21—H21A	119.8
C12—C7—S2	120.8 (2)	C23—C22—C21	120.4 (3)
C8—C7—S2	118.3 (2)	C23—C22—H22A	119.8
C9—C8—C7	118.1 (3)	C21—C22—H22A	119.8
C9—C8—H8A	120.9	C22—C23—C24	119.6 (3)
C7—C8—H8A	120.9	C22—C23—H23A	120.2
C8—C9—C10	122.5 (3)	C24—C23—H23A	120.2
C8—C9—N2	118.3 (3)	C23—C24—C25	120.4 (3)
C10—C9—N2	119.2 (3)	C23—C24—H24A	119.8
C11—C10—C9	118.4 (3)	C25—C24—H24A	119.8
C11—C10—H10A	120.8	C24—C25—C26	120.2 (3)
C9—C10—H10A	120.8	C24—C25—H25A	119.9
C10—C11—C12	120.6 (3)	C26—C25—H25A	119.9
C10—C11—H11A	119.7	C21—C26—C25	119.0 (3)
C12—C11—H11A	119.7	C21—C26—C27	119.5 (3)
C7—C12—C11	119.4 (3)	C25—C26—C27	121.4 (3)
C7—C12—H12A	120.3	C26—C27—S4	105.9 (2)
C11—C12—H12A	120.3	C26—C27—H27A	110.6
C20—S3—C19	104.77 (14)	S4—C27—H27A	110.6
C20—N3—H1N3	120.0	C26—C27—H27B	110.6
C20—N3—H2N3	120.0	S4—C27—H27B	110.6
H1N3—N3—H2N3	120.0	H27A—C27—H27B	108.7
C20—N4—H1N4	120.0	N5—C28—N6	121.5 (3)
C20—N4—H2N4	120.0	N5—C28—S4	116.1 (2)
H1N4—N4—H2N4	120.0	N6—C28—S4	122.4 (2)
			
O5—S1—C1—C2	30.8 (3)	O7—N2—C9—C10	175.5 (3)
O4—S1—C1—C2	152.1 (2)	C8—C9—C10—C11	0.1 (4)
O3—S1—C1—C2	−90.1 (2)	N2—C9—C10—C11	−178.6 (3)
O5—S1—C1—C6	−153.3 (2)	C9—C10—C11—C12	−0.2 (4)
O4—S1—C1—C6	−32.0 (2)	C8—C7—C12—C11	0.1 (4)
O3—S1—C1—C6	85.8 (2)	S2—C7—C12—C11	179.0 (2)
C6—C1—C2—C3	−1.3 (4)	C10—C11—C12—C7	0.1 (4)
S1—C1—C2—C3	174.5 (2)	C18—C13—C14—C15	0.0 (5)
C1—C2—C3—C4	0.5 (4)	C13—C14—C15—C16	0.1 (5)
C2—C3—C4—C5	0.5 (4)	C14—C15—C16—C17	−0.2 (5)
C3—C4—C5—C6	−0.8 (4)	C15—C16—C17—C18	0.3 (5)
C3—C4—C5—N1	179.1 (2)	C16—C17—C18—C13	−0.2 (4)
O2—N1—C5—C6	5.8 (4)	C16—C17—C18—C19	−173.9 (3)
O1—N1—C5—C6	−174.1 (2)	C14—C13—C18—C17	0.1 (4)
O2—N1—C5—C4	−174.1 (3)	C14—C13—C18—C19	173.6 (3)
O1—N1—C5—C4	6.0 (4)	C17—C18—C19—S3	−135.9 (2)
C4—C5—C6—C1	0.0 (4)	C13—C18—C19—S3	50.5 (4)
N1—C5—C6—C1	−179.8 (2)	C20—S3—C19—C18	67.8 (2)
C2—C1—C6—C5	1.0 (4)	C19—S3—C20—N4	17.4 (3)
S1—C1—C6—C5	−174.9 (2)	C19—S3—C20—N3	−163.9 (2)
O9—S2—C7—C12	28.0 (3)	C26—C21—C22—C23	0.2 (5)
O8—S2—C7—C12	−92.5 (2)	C21—C22—C23—C24	0.1 (5)
O10—S2—C7—C12	148.9 (2)	C22—C23—C24—C25	−0.7 (5)
O9—S2—C7—C8	−153.1 (2)	C23—C24—C25—C26	1.0 (5)
O8—S2—C7—C8	86.4 (2)	C22—C21—C26—C25	0.1 (4)
O10—S2—C7—C8	−32.2 (3)	C22—C21—C26—C27	−178.5 (3)
C12—C7—C8—C9	−0.2 (4)	C24—C25—C26—C21	−0.7 (4)
S2—C7—C8—C9	−179.1 (2)	C24—C25—C26—C27	177.9 (3)
C7—C8—C9—C10	0.1 (4)	C21—C26—C27—S4	108.2 (3)
C7—C8—C9—N2	178.8 (3)	C25—C26—C27—S4	−70.4 (3)
O6—N2—C9—C8	177.2 (3)	C28—S4—C27—C26	−159.2 (2)
O7—N2—C9—C8	−3.3 (4)	C27—S4—C28—N5	160.8 (2)
O6—N2—C9—C10	−4.0 (4)	C27—S4—C28—N6	−18.8 (3)

### Hydrogen-bond geometry (Å, °)

**Table:**

*D*—H···*A*	*D*—H	H···*A*	*D*···*A*	*D*—H···*A*
N3—H1N3···O4^i^	0.86	1.97	2.796 (3)	162
N3—H1N3···S1^i^	0.86	2.84	3.694 (3)	170
N3—H2N3···O3^ii^	0.86	2.05	2.898 (3)	171
N4—H1N4···O3^i^	0.86	2.26	3.080 (3)	160
N4—H2N4···O5^iii^	0.86	2.37	3.126 (3)	146
N5—H1N5···O10^i^	0.86	1.91	2.764 (3)	176
N5—H1N5···S2^i^	0.86	2.85	3.642 (3)	154
N5—H2N5···O9^ii^	0.86	1.94	2.783 (3)	168
N6—H1N6···O1^iii^	0.86	2.27	3.072 (3)	156
N6—H2N6···O8^iii^	0.86	2.07	2.787 (3)	141
C6—H6A···O4	0.93	2.56	2.900 (3)	102
C8—H8A···O10	0.93	2.57	2.896 (4)	101
C19—H19B···O4^iii^	0.97	2.51	3.331 (4)	142
C27—H27B···O8^iii^	0.97	2.53	3.259 (4)	132

Symmetry codes: (i) *x*+1, *y*+1, *z*; (ii) *x*, *y*+1, *z*; (iii) *x*+1, *y*, *z*.
